# Robotic Versus Laparoscopic Low Anterior Resection: Comparison of Peri-Operative Outcomes at United Lincolnshire Teaching Hospitals NHS Trust

**DOI:** 10.7759/cureus.96572

**Published:** 2025-11-11

**Authors:** Nadeem Ahmad Bhat, Rajalakshmi Venkateswaran, Nuha Amri, Trisha Kanani, Athula Tennakoon, Amit Shukla, Sridhar Dharmavaram

**Affiliations:** 1 General and Colorectal Surgery, United Lincolnshire Hospitals NHS Trust, Lincoln, GBR; 2 General Surgery, United Lincolnshire Hospitals NHS Trust, Lincoln, GBR; 3 Hepato-Pancreato-Biliary Surgery, University Hospitals of Leicester NHS Trust, Leicester, GBR

**Keywords:** anastomotic leak, laparoscopic lar, neoadjuvant chemo - radiation, rectal cancer, robotic assisted low anterior resection

## Abstract

Aim

The aim of this study was to compare the intra-operative and post-operative outcomes of robotic-assisted low anterior resection (LAR) and laparoscopic LAR.

Materials and methods

The study was a retrospective one, with the study period being January 2021 to December 2024. It was conducted across three hospitals of the United Lincolnshire Teaching Hospitals NHS Trust, United Kingdom. All patients diagnosed with an operable rectal cancer were included in this study. Data was collected from the case records of all patients. Besides demographic data, the pre-operative parameter considered was whether the patient received neoadjuvant chemoradiation. Intra-operative parameters analysed were operative duration, whether the procedure was converted to open, if a stoma was created, and the presence of any specific intra-operative complications. Length of stay in hospital and incidence of post-surgical complications, such as paralytic ileus, small bowel obstruction, anastomotic leak, bowel perforation, repeat surgery, hemorrhage or hematoma formation, sizable intra-abdominal collections, etc., were documented and analysed. Lastly, histopathological outcomes, the number of lymph nodes harvested, and the circumferential resection margins were analysed.

Results

Group A comprised 37 patients who underwent robotic LAR, and Group B comprised 48 patients who underwent laparoscopic LAR. The mean intra-operative duration (475±20.96 minutes for Group A versus 447.29±29 minutes for Group B) between the two groups showed a statistically significant difference (p value = 0.001). The chi square test showed better intra-operative and post-operative outcomes for Group A patients, with statistically significant differences, in the number of patients needing a de-functioning ileostomy (p = 0.001), rates of conversion to open surgery (p = 0.041), average lymph nodes harvest (p = 0.05), anastomotic leak (p = 0.03), paralytic ileus (p = 0.02), and re-laparotomy (p = 0.03). The unpaired t-test showed a significant difference between the average circumferential resection margins between Group A and Group B (degree of freedom (df) = 83, critical value = 1.99, and t = 5.1272) and the average duration of hospital stay (df = 82, critical value = 1.99, t = 2.97, further highlighting improved outcomes in Group A patients.

Conclusion

The three-dimensional magnified vision of the robotic platform, precise instrumentation, and the enhanced dexterity of the robotic arms help perform a precise dissection in the mesorectal fascial plane, achieve better tumor-free resection margins, and complete resection of the disease. The improved vision allows for resection of the disease while preserving the blood supply of the anastomotic ends, thus facilitating better clinical outcomes and early discharge from the hospital.

## Introduction

The incidence of colorectal cancers in the United Kingdom (UK) has been reported as 42,000 cases per year by Cancer Research UK, with around 16,000 deaths attributed to it [1].

Mid to low early rectal cancers are amenable to complete treatment with a combination of surgical resection and chemoradiation. The type of surgical approach for a low rectal cancer can have a bearing on the quality of surgery as well as the outcomes. The widely practised laparoscopic low anterior resection for low rectal cancers can be technically challenging, especially in the case of a narrow pelvis, post-neoadjuvant chemoradiotherapy, or prior irradiation for other cancers like prostate cancer. Robotic-assisted low anterior resection is believed to overcome at least some of these challenges by providing improved dexterity, precise instrumentation, and high-definition three-dimensional (3D) visualisation.

The surgical procedure done for mid to low rectal cancers (5-6 cm from the anal verge) is usually a low anterior resection (LAR). The procedure involves mobilization of the sigmoid, the descending colon [2], along with mobilisation and resection of the rectum at least 1 cm beyond the margins of the tumour [3], adequate vascular control [2], and a good total mesorectal excision (TME) [2]. Iatrogenic rectal perforation, ureteric injury, anastomotic leakage, low anterior resection syndrome, bladder injury, and urinary and sexual dysfunction are amongst some notable complications of this procedure [4]. Minimal invasive surgery (MIS) has superseded open resection in minimizing most of the aforementioned complications in rectal surgery; however, they still occur in laparoscopic as well as robotic-assisted rectal surgeries.

This study aims to compare the two MIS approaches, namely laparoscopic LAR and robotic-assisted LAR, in terms of certain intra-operative and post-operative outcomes of clinical significance.

## Materials and methods

This was a retrospective study conducted across three district hospitals of the United Lincolnshire Teaching Hospitals NHS trust in the United Kingdom, namely Lincoln County Hospital, Grantham and District Hospital, and Pilgrim Hospital Boston. The data collection period was June 2021 to December 2024.

All patients diagnosed with a mid to low rectal cancer and deemed operable (with an operable mid to low rectal cancer) following colorectal multidisciplinary team (MDT) discussion were included in this study. The exclusion criteria included patients less than 18 years of age, metastatic rectal cancers, and inoperable cases post neoadjuvant chemotherapy. Patients who underwent robotic-assisted resection of the rectal tumors were categorised as Group A, and patients who underwent laparoscopic surgery for the same were categorized as Group B. All patients were operated on by consultant colorectal surgeons with over five years of experience in the specialty. The robotic platform used for all the procedures in this study was the Da Vinci Xi Robotic Surgical System (Intuitive Surgical, Inc., Sunnyvale, California, United States).

Case records of all patients were assessed for pre-operative, intra-operative, and post-operative parameters. Besides demographic data, the pre-operative parameter analysed included whether the patient received neoadjuvant chemoradiation or not. Intra-operative parameters analysed were operative duration, whether the procedure was converted to open, if a stoma was created, and the occurrence of any specific intra-operative complications. Length of stay in hospital and incidence of post-surgical complications, such as paralytic ileus, small bowel obstruction, anastomotic leak, bowel perforation, repeat surgery, hemorrhage or hematoma formation, sizable intra-abdominal collections, etc., were part of the post-operative data documented and analysed. Histopathological outcomes of the two resection groups, which included the number of lymph nodes harvested and the circumferential resection margins, were also studied.

The data collected were entered into Microsoft Excel (Microsoft Corporation, Redmond, Washington, United States) and analyzed using IBM SPSS Statistics for Windows, version 21 (IBM Corp., Armonk, New York, United States). The chi-square test and unpaired t-test were used to analyze the variables, and a p-value of <0.05 was considered significant.

## Results

A total of 85 patients were included in the study. Group A comprised 37 patients (22 female and 15 male patients) who underwent robotic LAR, and Group B comprised 48 patients (21 female and 27 male patients) who underwent laparoscopic LAR. The average age of Group A and Group B patients was 72.24±6.98 years and 71.37±8.16 years, respectively. Twelve patients of Group A and 25 patients of Group B received neoadjuvant chemotherapy prior to surgery. 

Group A and Group B had mean operative times of 475.24±20.96 and 447.29±28.83 minutes, respectively, with the unpaired t-test showing a t value of 4.911 and a p value < 0.001. The number of patients who had a de-functioning loop ileostomy at the end of the surgery is given in Table 1, with the chi-square test showing a statistically significantly higher rate of stoma creation in Group B (p value = 0.001). The rates of conversion to open surgery are as depicted in Table 1, with a significantly higher rate of conversion to open surgery in Group B (p value = 0.04). 

**Table 1:** Comparison of rates of creation of defunctioning loop ileostomy and conversion to open surgery. Group A comprised patients who underwent robotic low anterior resection, and Group B comprised patients who underwent laparoscopic low anterior resection

Parameter	Group A (n= 37)	Group B (n=48)	Chi-square test value	p value
Creation of defunctioning loop ileostomy	18	39	10.054	0.001
Procedure converted to open	0	5	4.095	0.041

A comparison of the rates of post-surgical complications amongst Group A patients and Group B patients is given in Table 2.

**Table 2:** Comparison of post-surgical complications between the two study groups Group A comprised patients who underwent robotic low anterior resection, and Group B comprised patients who underwent laparoscopic low anterior resection

Parameter	Group A	Group B	Chi square test value	p value
Small Bowel Obstruction (SBO)	2	5	0.69	0.40
Anastomotic leak	1	8	4.303	0.03
Paralytic ileus	5	17	5.226	0.02
Intra - abdominal collection	1	4	1.197	0.2
Intra- abdominal hematoma/ hemorrhage	1	5	1.896	0.16
Small bowel perforation	0	1	0.78	0.37

The chi-square test showed a significantly lower rate of anastomotic leak and paralytic ileus amongst patients of Group A as compared to Group B, with p-values of statistical comparison of these variables being 0.03 and 0.02, respectively. The incidence of other complications (small bowel perforation (SBO), intra-abdominal collection, and intra-abdominal hematoma), despite being higher in Group B, was not significantly higher when compared using tests of significance. The chi-square test revealed that the return-to-theatre numbers in Group B were significantly higher when compared with Group A (10 versus 2, respectively; p value = 0.03). Of the 10 patients from Group B taken for a repeat laparotomy, three had an anastomotic leak, two had an acute SBO, three had a negative laparotomy, one had a moderate hemoperitoneum, and one had a jejunal perforation. The histopathological analysis report of the resected specimen in each case was analysed for the status of the circumferential resected margins (CRM) and the number of lymph nodes harvested in each case. A computed average of the aforementioned variables and the values of their respective tests of significance is given in Table 3.

**Table 3:** Comparison of histological findings amongst the two groups Group A comprised patients who underwent robotic low anterior resection, and Group B comprised patients who underwent laparoscopic low anterior resection CRM: circumferential resected margins

Parameter	Group A	Group B	p value	t value
CRM (millimeters)	7.24+/- 2.33	4.59 +/- 2.70	< 0.05	5.1272
Average number of lymph nodes harvested	17.02 +/- 5.07	12.10 +/- 3.25	< 0.05	4.7486

When the CRM averages amongst the two groups were compared, the unpaired t-test revealed a statistically significant difference in the means of both groups (degree of freedom (df) = 83, critical value = 1.99, t value = 5.1272). The unpaired test also revealed a statistically significant difference in the averages of the lymph node harvest amongst the two groups (df = 83, critical value = 1.99, t value = 4.7486). 

The average duration of hospital stay was higher in Group B as compared to Group A (9.02±5.74 days versus 5.7±3.77 days, respectively), with the longest stay duration being 33 and 21 days in Group B and Group A, repectively. The unpaired t-test showed a statistically significant difference between the two averages (df = 82, critical value = 1.99, t = 2.97). The mortality rates were comparable, with one death in each group in a 30-day follow-up period (p value = 0.85).

## Discussion

Laparoscopic colorectal surgery was successfully performed for the first time in 1990 by Jacobs (reported in 1991) [5]. During 1991-2001, laparoscopic colorectal surgery outweighed open colorectal surgery in terms of having adequate tumor-free resection margins, decreased blood loss, early recovery after surgery, and minimal post-surgical complications, shorter hospital stays, less post-operative pain, and decreased incidence of paralytic ileus. However, there were limitations to laparoscopic colorectal surgery, especially with respect to rectal tumor resection. The two-dimensional (2D) vision and limited dexterity were identified as major limitations, especially in cases with a narrow pelvis, extensive fibrosis in the pelvis secondary to neoadjuvant chemoradiation, and dense adhesions.

Wang Chu and colleagues, in their study on 14 patients in 2014, performed an objective assessment of certain factors that increased operative time in laparoscopic rectal cancer surgery and concluded that high BMI and a narrow pelvis significantly prolonged the operative time (p value: 0.03) [6]. These factors did influence the occurrence of anastomotic leak, as predicted in various studies. Zu Kian et al., in their study on 132 patients, concluded that a tumor size > 3 cm, distance of 6 cm from the anal verge, and advanced TNM-staged tumours were independent risk factors for anastomotic leak post-surgery, with their relative risk being 1.149, 0.552, and 2.816, respectively [7].

Robotic-assisted surgery is thought to resolve these complications. The concept of robotic-assisted colorectal surgery first came into light only in 2001, and it was first reported in 2002 by Weber et al. for benign colorectal disease [8], and by Hashizume et al. for malignant disease [9]. 

Identifying the correct plane for TME can ensure an adequate tumor resection whilst evading several complications. Dissection in proper rectal fascial planes, taking precautions to avoid dissecting into the presacral and Waldeyer’s fascia, can help prevent injury to the pelvic autonomic nerves and veins [2]. This will ensure the bladder and sphincter functions are preserved and there is no severe hemorrhage. The enhanced 3D magnification view offered by the camera system and the enhanced dexterity of the robotic arms can help dissect into the correct avascular plane for a bloodless and precise TME. 

A systematic review in 2021 identified several risk factors for anastomotic leak in colorectal surgery. Male gender, previous chemo-radiation, high BMI (> 30 kg/m^2^), American Society of Anesthesiologists (ASA) grade > 2, smoking, malnourishment, type of approach to surgery, etc, have been identified as independent risk factors for an anastomotic leak after surgery [10]. While a few of these are modifiable factors, surgery for cancers cannot be postponed until these factors are reversed.

Neo-adjuvant chemoradiation is known to distort the anatomy of the planes for dissection [11,12]. Hence, in such cases where there are already known risk factors for an anastomotic leak, precision surgery can help reduce the effects of the technical causes for the failure of the anastomosis. Minimal hemorrhage, identification of the right plane, and precise dissection, which is better achieved by robotic surgery, allow for appropriate mobilisation of the colon, preservation of the blood supply of the anastomotic ends, and ensure the anastomosis is not under tension, thus minimising the risks for an anastomotic leak. Post-operative benefits include a lower incidence of paralytic ileus, lower post-surgical complications, and hence a faster discharge from the hospital. 

Matsuyama and colleagues conducted a study in 2021 in Japan comprising 5650 patients, divided equally into two groups, which showed that the robotic-assisted LAR group had a longer operating time (352 versus 283 minutes; p < 0.001), less intra-operative blood loss (15 versus 20 ml; P < 0.001), a lower in-hospital mortality rate (0.1% versus 0.5%; P = 0.007), and a shorter post-operative hospital stay (median: 13 versus 14 days; P < 0.001) compared with the laparoscopic LAR group [13]. These study results were further validated in our study. 

Kim et al., in their study, showed no significant difference in harvesting of lymph nodes amongst the two groups (20.5±9.9 vs 19.7±7.3, p = 0.753) [14]. However, our study had contradicting results, with the robotic surgery group having a significantly higher harvest of lymph nodes.

The current study showed lower rates of conversion to open, fewer patients with a defunctioning ileostomy, fewer post-surgical complications (11.66% in the robotic-assisted LAR group and 38.33% in the laparoscopic LAR group), and better CRM and lymph node harvest. This, in our opinion, was possible only because of the superior vision and the 7 degrees of freedom of the robotic arm that permits precise dissection. Feng et al. [15] and Hettiarachchi et al. [16] have confirmed similar results in their research papers as well. 

The drawbacks of robotic surgery are high costs, the requirement of experienced theatre staff, a steep learning curve, and unavoidable power failures that can result in recoverable and non-recoverable faults. As a result of this, it is still impossible to offer all patients robotic surgery. However, an appropriate selection of patients for both types of procedures can help in maximising favourable patient outcomes. Patients with raised BMI [14], previous history of neoadjuvant chemoradiation [15], and an irradiated pelvis due to other malignancies should be planned for robotic rectal surgeries, as studies have shown better clinical outcomes in these patients. (Albayati et al., in their study, have shown that the rates of conversion to open were significantly lower in morbidly obese patients (BMI> 35 kg/m^2^) undergoing robotic-assisted colorectal surgery (p value: 0.027) [17]. Zhang and colleagues published a meta-analysis in 2024 that showed robotic surgery significantly reduces the risk of conversion to open surgery (OR 0.46, 95%CI 0.40, 0.53) and improves the TME incomplete rate (OR 0.40, 95%CI 0.17, 0.93) in patients who have received neo-adjuvant chemoradiation [18]. 

The limitations of the study include a small study population and unequal study groups. It being a retrospective study, certain confounding factors could not be matched, which could have further deepened the scope of the dissection. Multicentric studies, an extended follow-up period, and a larger study population can help validate the studies.

## Conclusions

The 3D magnified vision of the robotic platform, precise instrumentation, and the enhanced dexterity of the robotic arms help perform a precise dissection in the mesorectal fascial plane, achieve better tumor-free resection margins, and complete resection of disease. The improved vision allows for resection of the disease while preserving the blood supply of the anastomotic ends, thus facilitating better clinical outcomes and early discharge from the hospital.
